# The ambiguities of Indonesian Sustainable Palm Oil certification: internal incoherence, governance rescaling and state transformation

**DOI:** 10.1007/s10308-020-00593-0

**Published:** 2021-01-18

**Authors:** Shofwan Al Banna Choiruzzad, Adam Tyson, Helena Varkkey

**Affiliations:** 1 Department of International Relations, University of Indonesia, Depok, 16424 Indonesia; 2 School of Politics and International Studies, University of Leeds, Leeds, LS2 9JT UK; 3 Department of International and Strategic Studies, University of Malaya, 50603 Kuala Lumpur, Malaysia

**Keywords:** Sustainable palm oil, Governance rescaling, State transformation, Indonesia, Europe

## Abstract

There are persistent tensions of both a technical and political nature between Southeast Asia’s two major palm oil producers, Indonesia and Malaysia, and the sustainability governance mechanisms shaping global environmental and trade standards emerging from Europe. The establishment of the national Indonesian Sustainable Palm Oil (ISPO) certification standard in 2011 is a sign of discontent with the transnational Roundtable on Sustainable Palm Oil (RSPO) regime, sparking debate about the legitimacy of private governance models initiated by non-governmental organizations and companies in Europe. This article questions whether the adoption of sustainability norms by Indonesia signals normative convergence or the emergence of rival governance structures that challenge the state. Evidence suggests that elements of norm adoption and rival governance coexist in Indonesia and that ISPO certification is an ambiguous policy with degrees of internal incoherence. The ambiguous nature of ISPO certification gives rise to unresolved disputes over power and authority between various actors. This article shows how these disputes came into being by framing these dynamics as part of a long historical process. Novel insights are gained by employing the state transformation framework and the concept of governance rescaling. Within this framework, we argue that the ambiguous nature of the ISPO results from complex interrelated processes of fragmentation, decentralization and the internationalization of the Indonesian state.

## Introduction

In January 2020 Indonesian President Joko Widodo (hereafter Jokowi) accused the European Union (EU) of provoking an unjust trade war that discriminates against palm oil production (Nathalia 2020). In 2020, Indonesia moved to bring the EU’s renewable energy directive (RED II) to the World Trade Organization’s Dispute Settlement Mechanism (DS593), attracting one of the highest rates of third-party participation in the history of the WTO. Indonesia’s response to EU claims of indirect land use change and negative externalities caused by palm oil production is often framed as tensions between developed and developing countries, or the contentious diffusion of global sustainability norms. We argue that this is only a part of the story.

This article contends that Indonesian state responses to criticisms of the palm oil industry are complex and nuanced. To illustrate this point, the authors examine Indonesia’s response to the 2004 Roundtable on Sustainable Palm Oil (RSPO), a transnational private governance mechanism for sustainable palm oil supported in principle by many EU-based companies and non-governmental organizations (NGOs).^1^ Facing growing international criticism for the social and environmental impacts of palm oil production, the government of Indonesia established the Indonesian Sustainable Palm Oil certification standard (hereafter the ISPO) in March 2011.^2^ As a potential rival to the RSPO, the ISPO raises questions about the authority and status of previously established multi-stakeholder voluntary international standards on sustainable palm oil. Statements from government officials in Indonesia are often ambiguous and do little to clarify the status of rival sustainability standards. For instance, Achmad Mangga Barani, the Director General of Plantations in the Ministry of Agriculture at the time, stated that the ISPO refers to RSPO’s principles and criteria and thus does not rival the RSPO (Lestari 2010), while the Minister of Agriculture from 2009 to 2014, Suswono, argued that the ISPO ‘has its own perspective which is different from other countries’ (Zuhri 2011). These statements begin to show that the establishment of the ISPO leads to conflicting interpretations of how the state-regulated certification scheme interacts with global and EU-led sustainability norms.

This article offers a comprehensive understanding of the ISPO by drawing upon the state transformation framework and the concept of governance rescaling. To better understand the ISPO, we examine the evolution of the Indonesian state and the development of the palm oil industry in the country, including the competition for governance rescaling that follows the transformation of the state in the late 1990s that enabled the establishment of the RSPO and then the ISPO. To elaborate on this central point, this article proceeds in five parts. Part one looks at the existing debate on the nature of ISPO and the relationship between RSPO and ISPO to provide the context in which this article aims to contribute. The second part examines the links between state transformation frameworks and concepts of governance rescaling to conceptualize the ISPO in the context of global trade, followed by an explanation of the data collection methods and evidence. Part three looks at the development of Indonesia’s palm oil industry, especially in the post-1998 democratic transition and liberalization era. Part four examines the establishment of the RSPO as a response from competing networks of actors to the rapid expansion of the-* palm oil sector and the continuous tension within the RSPO. Part five examines the establishment of the ISPO by framing it as part of the continuous transformation of the Indonesian state. The authors conclude that the internationalization of the state apparatus leads to the further fragmentation of the Indonesian state bureaucracy that renders the ISPO an ambiguous and contentious policy that is ineffective on two fronts: sustainability as well as industry protection.

## Research gap: ambiguities of the ISPO-RSPO relationship

There are divergent views on the relationship between the RSPO and the ISPO. By some measures, the establishment of the ISPO in 2011 indicates the proliferation and influence of global sustainability norms. EU normative power influences regulatory standards for global environmental and trade governance in line with renewable energy targets, though there are forms of institutional and networked opposition from Indonesia (Sicurelli 2020; Tyson and Meganingtyas 2020). While acknowledging that the ISPO has different characteristics from the RSPO, both are viewed as ‘initiatives to align oil palm production with the principles of sustainable development’ (Ernah et al. 2016). Both are perceived as part of the same trend in global supply chains to mainstream sustainability norms while transforming the market to create sustainable products (Hutabarat 2017).

An opposing viewpoint is that the ISPO is a state-driven rival governance mechanism to counter the RSPO. The ISPO appears to show the ambition of the Indonesian state to reclaim authority from the market-driven RSPO (Giessen et al. 2016), with the ISPO representing ‘an expression of government sovereignty’ (Astari and Lovett 2019). Indeed, the ISPO may represent an effort by the global South to resist interventions from the North (Hospes 2014; Schouten and Bitzer 2015; Higgins and Richards 2019). In this view, the ISPO is an attempt to reframe sustainable palm oil to enable the palm oil sector in Indonesia to bypass the RSPO to more effectively access global markets (Higgins and Richards 2019, 126). In contrast to the RSPO which seeks legitimacy by relying on external audiences such as non-governmental organizations (NGOs) and companies in developed countries who do not themselves implement standards, the ISPO relies on internal processes of legitimization with national producers and trade associations as the focal audience. The establishment of the ISPO is also a refutation to the claim of a governance void employed by western-influenced global standard setters such as the RSPO (Schouten and Bitzer 2015).

Given the opposing views on the ISPO, this article argues that both the adoption of global sustainability norms and the rival governance arguments are partially correct. This article finds that the ISPO is an ambiguous policy, attempting to restore the primacy of the national domain of governance and to provide more room for the domestic palm oil industry while simultaneously internalizing the norms promoted by transnational private governance. There is a continuous tension within the ISPO itself, which makes it internally incoherent. This is evidenced by the complex and uneven responses of the domestic palm oil industry to the certification initiative. For instance the influential Indonesian Palm Oil Association (GAPKI) urges the government to use the ISPO as a diplomatic instrument to overcome trade barriers against the commodity (GAPKI 2017). At the same time, GAPKI contends that the stringent implementation of the ISPO would potentially harm the industry (Supriyanto and Hidranto 2014). With this ambiguity, the ISPO failed to meet the expectations of both reformists who want to improve sustainable palm oil governance and protectionists who want to use the ISPO to protect the palm oil industry from negative media coverage and trade discrimination.

Studies about the nature of the ISPO and its connection to different public and private actors in Indonesia and beyond have touched upon these ambiguities directly and indirectly, although tend not to explicitly identify the ambiguity of the ISPO (Suharto et al. 2015; Giessen et al. 2016; Hidayat et al. 2018; Nesadurai 2018; Astari and Lovett 2019; Higgins and Richards 2019). In a study of the nature of the ISPO that conceptualizes the palm oil regime complex, Pacheco et al. (2020) contend that there are both complementarities and antagonisms between state regulations such as the ISPO and international standards such as the RSPO. Pacheco et al. (2020) see that the palm oil sector is increasingly governed by a transnational regime complex involving state policies and regulations, market-based mechanisms and self-regulatory initiatives that interact on different scales, from the global to the subnational. Important complementarities between state regulations and market-driven initiatives are visible at the international level, while there are emerging ‘disconnects and antagonisms’ at national and subnational levels (Pacheco et al. 2020, 582).

A study by Pramudya, Hospes and Termeer (2018, 17) touches upon the ambiguity of the ISPO, observing that the Indonesian state responses to transnational private governance efforts such as the RSPO are unstable and changing dynamically. Tracing the development of the government of Indonesia’s response to the RSPO, including the establishment of the ISPO, there is a continuous evolution from co-optation by non-state actors to coordination, from coordination to competition and then from competition back to coordination again, although in a much more limited manner (Pramudya et al. 2018, 11). This critical ambiguity is arguably the result of ‘tension between formulating stricter regulations on the environmental impacts of the expansion of palm oil plantations and the Indonesian government’s main target of economic expansion of the sector’ (Hidayat et al. 2018, 234).

Evidence suggests that elements of norm adoption and rival governance coexist in Indonesia and that ISPO certification is an ambiguous policy with degrees of internal incoherence. The ambiguous nature of ISPO certification gives rise to unresolved disputes over power and authority between various actors. This article shows how these disputes came into being by framing these dynamics as part of a long historical process. Novel insights are gained by linking the state transformation framework to the concept of governance rescaling. Within this framework, we argue that the ambiguous nature of the ISPO results from complex interrelated processes of fragmentation, decentralization and the internationalization of the Indonesian state.

## State transformation and governance rescaling

Concepts of state transformation and governance rescaling explain the ambiguity of ISPO as the Indonesian state response to transnational private governance. These approaches help locate and connect the development of the RPSO and the ISPO to historical trajectories of Indonesia’s political economy. State transformation frameworks explain state behaviour by understanding that states are in continuous transformation, which represent changes in political economy relations within and beyond the state (Hameiri and Jones 2016; Jones 2019). By seeing the state this way, we understand that some policies are internally incoherent and ambiguous because they are the temporary results of competition between various social forces. This approach sees states not as unitary actors and politically neutral problem solving instruments but as complex and fragmented ‘institutional ensembles that reflect and embed historically evolving social relations and competition for the distribution of power and resources’ (Hameiri and Jones 2016, 78). In Indonesia, state transformation results in a complex local-central dynamic which also affects the development of its regional and sub-regional cooperation projects (Karim 2019).

According to the state transformation framework, governance can be rescaled to new levels following the evolution of social conflicts because national governance is a socially and politically constructed historical artefact (Hameiri and Jones 2016, 79). Different scales of governance privilege different groups, and social forces are continuously struggling to define a scale of governance that most benefits them. The establishment of the RSPO and then the ISPO can be explained through this lens of governance rescaling. The RSPO is understood as an effort by global consumer companies based in the developed world to rescale governance from national to transnational levels as a way to convince environmentally aware consumers who, informed by various transnational advocacy groups, distrust the ability of the state in countries such as Indonesia to ensure sustainability practices in the palm oil sector. Domestic trade associations and businesses who control the palm oil industry in Indonesia respond negatively to rescaling efforts because it would mean less privilege for them to exploit. By framing their interests as the national interest and claiming sovereignty, the Indonesian palm oil sector is demanding support from the state to escape the control of the RSPO without losing market share.

Accelerated processes of decentralization, liberalization and state fragmentation in the post-1998 transitional era increased the entanglement between different parts of the Indonesian state apparatus and various transnational actors. In this context, some parts of the state bureaucracy internalized international norms such as sustainability and sought to reform the palm oil sector. Complex interactions between state bureaucracies seeking to reclaim their authority, the domestic industry seeking to protect their business and well-connected NGOs seeking to reform the palm oil sector result in the ambiguity of the ISPO.

Evidence for this study comes from interviews and observations conducted periodically in Indonesia from 2015 to 2020, crosschecked with a wide range of written sources including statements, in-house publications, minutes of meetings, press releases and position papers from the RSPO, the ISPO, palm oil companies, trade associations such as GAPKI and the palm oil growers’ association (APKASINDO), government sources such as the Ministry of Agriculture, the Ministry of Trade, the Ministry of Foreign Affairs and the Office of the President, as well as civil society actors. The authors analysed selective Indonesian news coverage of the palm oil industry from 2009 to 2020, mainly from sources such as *Bisnis Indonesia*, *Kompas* and *Republika* that focus on economic issues and have a wide national readership. The year 2009 marks the establishment of the first iteration of the EU’s renewable energy directive (RED I) and the start of a new era of export risk and contract cancellations. These written sources provide the plot and storyline to be connected and framed through theories of state transformation and governance rescaling.

The authors conducted in-depth interviews during different stages of fieldwork in Indonesia (Jakarta, Riau, Bandung, Surabaya) and the UK from 2015 to 2020. Interviewees were purposively chosen based on their expert knowledge of the palm oil industry and agribusiness in Indonesia, especially on the evolution of the RSPO and the ISPO. Semi-structured anonymized interviews were conducted so that participants could express their opinions freely. To obtain a comprehensive picture, the authors sampled elite informants as well as farming communities in Riau, Indonesia’s main palm oil producing province (Apresian et al. 2020).

Not all statements from interviewees are quoted directly in this article, but the cumulative insights we have gained over time guide the construction of the argument in this article. The next section elaborates on the dynamic processes of state transformation and governance rescaling (and counter-rescaling) in the Indonesian palm oil sector. To make it easier to follow, the storyline is presented in a semi-chronological manner.

## Key developments in the palm oil industry

While industry actors often proclaim that palm oil is ‘a gift from God for Indonesia’ (Supriyono 2016), the original habitat of the oil palm tree is in West Africa (Berger and Martin 2000, 397). It was the Dutch who first brought oil palm trees to the Bogor Botanical Gardens in Buitenzorg in 1848. The use of palm oil for industrial purposes began in the mid-nineteenth century in Central Java (Larson 1996), and the first commercial oil palm plantation was established in 1911 (PASPI 2014). The colonial Dutch East Indies quickly became one of the major global producers of palm oil. By 1937 it was the world’s largest exporter of crude palm oil (CPO), producing around 40% of total global exports according to the Palm Oil Agribusiness Strategic Policy Institute (PASPI 2014, 11). The complexity of post-colonial state-building after 1945, and President Sukarno’s nationalist economic policies that discouraged foreign investment and exports, led to the decline of palm oil production. In 1958 CPO exports fell to 17% of global market share (PASPI 2014, 11).

In the aftermath of a national political crisis and regime change in 1966, the authoritarian developmental New Order regime led by President Suharto revitalized the palm oil industry. Government estates that produced palm oil expanded from 84,000 ha in 1969 to 343,000 ha in 1987 due to direct government investment through the Perseroan Terbatas Perkebunan state owned plantations (Larson 1996; PASPI 2014). The growth of the palm oil industry in Indonesia was supported by the World Bank, which saw palm oil as an instrument for rural development and potential export commodity. In the late 1980s, the industry was gradually liberalized with the introduction of the People’s Nucleus Estate (Perkebunan Inti Rakyat, PIR) model. Under the PIR model, the government facilitated more participation from the private sector by for instance awarding logging rights and concessionary rate credits, with the requirement that estates create partnerships with smallholders in their proximity (Larson 1996). These policies resulted in the growth of the industry, as the total plantation area reached 3.9 million ha by the late 1990s. Indonesia’s CPO production rose from a mere 188,000 t in 1969 to 6.4 million t in 1999 (PASPI 2014).

The 1997 Asian Financial Crisis heralded a political crisis that precipitated the fall of President Suharto in May 1998 and the IMF-led restructuring of Indonesia’s political and economic system. Institutional reforms that included liberalization and the further opening of the economy provided a boost for the palm oil industry (Sato 2003). Three main factors explain the emergence of Indonesia as the largest producer of palm oil in the world, overtaking Malaysia in 2006. The first factor is the opening up of the palm oil sector to foreign investment, which led to the mobilization of capital from regional players in Malaysia and Singapore. The Letter of Intent signed by the IMF and Indonesia on 15 January 1998 encouraged foreign investment. Accordingly, the government decided in June 1998 to issue a revised and shortened negative list of activities closed to foreign investors. As part of this process, the government removed restrictions on foreign investment in palm oil plantations in February 1998, while those on wholesale and retail trade were lifted in March 1998 (IMF 1998). The easing of restrictions on foreign investment in the palm oil sector provided incentives for companies in Malaysia and Singapore, which have abundant capital but limited land, to invest in Indonesia and increase production by controlling more plantation areas in Indonesia.

The second factor explaining the palm oil production boom is Indonesia’s political and administrative transformation. The promulgation of Law No. 22/1999 on Regional Government transferred many authorities from central to local governments, including the authority to issue plantation permits for palm oil. The third factor is global economic trends such as the rise of China and India that increased demand for palm oil (Ewing 2011). Following the flow of capital from Malaysia and Singapore to Indonesia, the increasing global demand was mostly met by CPO produced in Indonesia. Key indicators of governance rescaling such as liberalization and the opening of the palm oil sector to foreign investment mean further transformation from a developmental to a regulatory state that aims for global competitiveness. It also means the further transfer of authority from state to market mechanisms. Complex governance rescaling results in decentralization and fragmentation. As Resosudarmo (2005) notes, decentralization and the reluctance of central government to fully implement reforms causes vertical conflicts between different levels of government, as well as horizontal conflicts between local governments and power competition between government agencies. This is particularly evident in the palm oil sector, which has become a site of contestation between various social, political and economic forces.

Indonesia’s rapidly growing palm oil sector in the 2000s is strongly connected to state transformation. Fragmented authorities within the Indonesian state, marked by overlapping policies and regulations between central and local governments and among government agencies, has paradoxically created a conducive environment for growth. The expansion and regionalization of the industry has been driven by patronage politics (Varkkey 2012), which operates under a system of multilevel (mis)governance (Hamilton-Hart 2015). The 1999 decentralization policy shifted the authority to issue palm oil plantation permits to local governments, leading to a rise in the number of plantation permits in Sumatra and Kalimantan. Irregular spatial planning led to overlapping regulations and jurisdictions, resulting in permits being issued without proper procedures. Palm oil expansion has been strongly connected to corruption linked to campaign finance, and in the heavily cultivated province of Riau, three former governors have been jailed for corruption. Annas Maamun, the Governor of Riau from 2014, could not complete his term because he was caught by the Corruption Eradication Commission in a bribery case. He is guilty of accepting bribes to convert the land status of some oil palm plantations in Kuantan Singingi, Rokan Hilir and Bengkalis districts from forest to non-forest zones to legitimize production (Dipa 2015).

The factors enabling the rapid growth of the industry also inhibit the mechanisms to control its adverse effects (Hamilton-Hart 2015, 179). Responding to this situation, communities, often supported by local and international NGOs, engage in collective mobilization and criticize the business practices of palm oil companies. In Kalimantan, for example, rural women in Sambas mobilized against palm oil company PT SAM to defend their land (Morgan 2017). Protests against palm oil companies in Indonesia are documented in the 2018 film *Asimetris* directed by Dandhy Laksono and produced by WatchDoc.^3^ These key developments in the palm oil industry are precursors to the establishment of the RSPO and the ISPO, as examined in the next sections.

## The RSPO and the transition to sustainable palm oil

Forms of EU normative power are evident in the search for cleaner production and sustainable sourcing of palm oil. International NGOs often target the consumers of global brands that use palm oil as their materials, especially in Europe. The focus on consumer awareness in developed countries seems to be driven by surveys consistently showing that people in the EU and elsewhere value the environment and biodiversity. For instance, the European Commission conducted several Eurobarometer surveys on biodiversity (Flash Eurobarometer 2007; 2010; 2013; Special Eurobarometer 2015). The data consistently shows that most Europeans think that biodiversity loss is a serious issue and that they will be personally affected. The Eurobarometer Survey on the Attitudes of European Citizens towards the Environment found that the environment ‘has an indisputable importance in the lives of Europeans’, as 96% of respondents feel that protecting the environment is important for them personally (Special Eurobarometer 2008).

To mobilize consumers in developed countries, NGOs use various ‘naming and shaming’ campaigns. The use of opprobrium is designed to influence palm oil production by disrupting the end of the supply chain. The expectation is that market mechanisms will force producers to limit the harm done to the environment. For instance, in the early 2000s, the World Wildlife Fund (WWF) ran a lipstick from the Rainforest awareness campaign, followed shortly after by a Greenpeace campaign to hold the palm oil industry to account. A 2007 Greenpeace report entitled *How the Palm Oil Industry is Cooking the Planet* claims that the palm oil industry is the main contributor to deforestation in Indonesia, with 1.8 billion t of greenhouse gas (GHG) emissions being released annually. The report accuses global companies that source palm oil from Indonesia such as Unilever, Nestlé and Procter & Gamble of being complicit with environmental crimes (Greenpeace 2007). Unilever and its suppliers have been labelled ‘destroyers’ of forests and peatlands in Indonesia (Greenpeace 2008), while Nestlé, another major user of palm oil, is said to benefit from the burning of tropical forests and the loss of orangutan habitat (Greenpeace 2010).

As consumer awareness about the environmental impacts of the palm oil industry rises in developed countries, so does the pressure placed on global brands which use palm oil in their products. In May 2008, Unilever responded to pressure by declaring its commitment to clean up the company’s supply chain (Unilever 2008). In December 2009, the company cancelled a US$ 30 million contract with Golden Agri-Resources Limited (GAR) in Indonesia because of unsustainable practices (Gray 2011). In March 2010 Kraft, another major global brand followed suit (Adnan 2010). In the same year, the global fast food chain Burger King halted their cooperation with the Indonesian palm oil company (Kompas 2010). Unilever now has a 100% sustainably sourced palm oil pledge and has developed new geolocation monitoring technologies to improve supply chain transparency, whereas Sime Darby has a new open access Crosscheck 2.0 online traceability tool to support its goals of a deforestation-free supply chain.

Intensive NGO campaigns and market pressures create a dilemma for producers and the companies that use palm oil in their products. Companies need a steady supply of CPO for their business. Replacing palm oil with rapeseed or soybean oil produced in Europe is challenging because palm oil is generally a more cost-effective and high yielding flex-crop. The question for companies is how to convince conscientious consumers to buy their products while maintaining a steady supply of palm oil. In this context, transnational actors such as international NGOs and multinational companies based in developed countries, especially in Europe, agreed to initiate the Roundtable on Sustainable Palm Oil (RSPO).

Transnational private governance in the form of the RSPO has emerged as a significant benchmark for standards of sustainable palm oil, setting out key principles and criteria and developing a system for trade in certified sustainable palm oil (Hai 2013, 22). The number and composition of members, as well as the market impact of certified products to the supply chain, are important parts of the RSPO’s legitimacy, although this rests on an uneasy consensus between antagonistic groups with different interests and concerns, and the power asymmetries within the RSPO may erode its legitimacy. While the secretariat of the RSPO is in the Malaysian capital Kuala Lumpur, the RSPO is largely dominated by companies and NGOs based in Europe. The apparent domination of actors from developed countries continues to challenge the legitimacy of the RSPO, whose standards and best practices are benchmarked against those set by the UK-based ISEAL Alliance. Indonesian Palm Oil Association (GAPKI) officials often complain about the influence of developed countries that comes at the expense of producer countries. During a 2014 interview in Jakarta, an official from GAPKI suggested that ‘the fact that the RSPO was established in Switzerland shows who they are’. The composition of RSPO membership underrepresents producer countries. As of November 2020, from a total of 4941 members (including ordinary, affiliate and associate members), only 190 are palm oil producers. Neither Indonesia nor Malaysia are in the top ten countries ranked by RSPO membership (RSPO 2019a), creating some distrust towards the RSPO.^4^ For instance, GAPKI formally withdrew from the RSPO in September 2011.

The dominant role of Western NGOs and downstream actors in the palm oil supply chain in the RSPO is evident in the standards-making process. The RSPO works by establishing a standard for sustainable palm oil and creating a system to ensure compliance. As a roundtable, the standard conceptually should come from all the members. While formally this is true as all members have a voice in the process, the standard itself is set by developed countries. The proposed sustainability standard and adaptation strategy that were discussed in the preparatory meetings for the establishment of the RSPO in September 2002 in London were shaped by previous initiatives in Europe. In 1998 for instance, Unilever started to develop the indicators for sustainable palm oil, while in 2000 Migros, with the help of the WWF and Proforest, created supply chain standards that would form most of the RSPO’s principles (Nikoloyuk et al. 2010, 62).

Since 2004 the roundtable has been a site of contention between social forces with different interests and concerns. While both NGOs and companies in the northern hemisphere aim for governance rescaling through the RSPO, they have different priorities. NGOs strive for governance rescaling to mitigate the negative environmental and social impacts of the palm oil industry’s unsustainable practices. They advocate stricter principles and enforcement criteria for sustainability standards. Companies on the other hand need to balance their ledgers. They must keep their consumers satisfied while at the same time ensuring that the supply of affordable palm oil is not disrupted.

For NGOs the goal is stricter sustainability criteria and a stronger rules-based system of enforcement. This prompted the formation of the Compensation Task Force in 2011, which is generally seen by producers as changing the voluntary nature of the RSPO. NGOs such as the WWF insisted on bringing the draft of the guidance for compensation to the RSPO Executive Board in July 2013 despite objections from some palm oil producers. As a response, some RSPO members protested and walked out of the Executive Board meeting (RSPO 2013). NGOs within the RSPO continue to insist that the RSPO is not doing enough, echoing the criticisms from non-member NGOs operating outside of the RSPO. Greenpeace, for example, harshly criticized the RSPO as ‘certifying destruction’ (Greenpeace 2013). NGO members continue to argue that the RSPO is at risk of growing weaker. The Dutch-based NGO Aidenvironment, an RSPO member, threatened to withdraw after the RSPO revoked its suspension of the IOI Corporation for illegal forest clearance and planting on peatlands in West Kalimantan, Indonesia (Chow 2016). Aidenvironment decided to remain in the RSPO only when the IOI Corporation agreed to abandon 434 ha of ‘overplanted’ areas (Chin 2016), a small but apparently effective compromise. A Swiss NGO called PanEco to quit the RSPO in 2016, citing the ‘sheer level of inaction’ against the breach of RSPO principles by PT Sisirau, a company accused of destroying orangutan habitat. PanEco also criticized a new RSPO resolution that members should ‘promote and not denigrate’ certified sustainable palm oil despite its clear flaws (Jacobson 2016). This shows that NGOs both insider and outside the RSPO remain highly critical of the RSPO as a form of transnational private governance.

Companies at the downstream end of the palm oil supply chain, mostly based in developed countries, are also facing uncertainties. There are some true reformers within the industry seeking to change the way business in the palm oil sector is conducted. However, critics suspect that companies join the RSPO to enhance their brand image and reputation. Even Unilever, often hailed as the world’s leading champion of sustainability, is criticized for not being fully committed to sourcing identity preserved palm oil from genuinely sustainable plantations. Despite the company’s claims from its marketing division that it sourced all of its palm oil sustainably since 2012, critics pointed out that in 2015 only 19% of the palm oil actually came from RSPO-certified plantations (Dupont-Nivet et al. 2017). In response, Unilever published its 2016 Sustainable Palm Oil Sourcing Policy that commits the company to 100% physically certified palm oil and its derivatives (Unilever 2016). The case of Unilever illustrates that companies can use the RSPO as a branding platform to boost their markets despite ongoing uncertainty about sustainable practices in their supply chain.

Criticisms of downstream companies are not only coming from NGOs. Producers accuse downstream companies of being unduly influenced by networks of NGOs while at the same time being unwilling to share the burdens of sustainability. While growers are forced to fulfil RSPO principles and criteria to produce certified sustainable palm oil, downstream companies push producers to certify their palm oil and raise standards but do not actually purchase large quantities of certified sustainable palm oil. In 2018, for example, from a total supply of 13,287,565 metric tons of certified CPO, less than half was actually sold (RSPO 2019b). As a Bloomberg report aptly puts it, the world has an abundance of sustainable palm oil, ‘but no one wants it’ (Raghu 2019). Some producers complain of the hypocrisy of downstream companies that cooperate with NGOs to make the certification process stricter, and thus more complicated and costly, out of concern for their brand image, while proving unwilling to pay a premium for the certified product.

Palm oil producers inside and outside the RSPO, especially those based in Indonesia, often accuse NGOs of not having genuine concerns about the environment and the negative social impacts of the palm oil industry. Some contend that the NGOs are part of a trade war designed to decrease the competitiveness of palm oil (Choiruzzad 2019). With the one member one vote system, producers feel that the RSPO is tilted to the interest of international NGOs and downstream companies. This perception led to the withdrawal of GAPKI from the RSPO, although many Indonesian companies that are also GAPKI members remain in the RSPO to secure their market (Merdeka 2011). Complex responses to the RSPO reflect concerns about the legitimacy of private governance models initiated by non-governmental organizations and companies in Europe. The 2011 establishment of the national Indonesian Sustainable Palm Oil (ISPO) certification standard is a manifestation of the discontent with the RSPO among some producers and government bodies.

## The ISPO as a counter-rescaling strategy

GAPKI’s withdrawal from the RSPO in 2011 coincides with the establishment of the ISPO by the Indonesian Ministry of Agriculture. Thus, despite being presented as compatible with the RSPO, many observers see the ISPO as a rival governance mechanism (Hospes 2014). This view seems consistent with the previous attitudes of Indonesian government officials regarding the palm oil controversy. When the palm oil boom in the 2000s resulted in criticisms from domestic and international NGOs as well as international actors such as the EU, the standard response has been to defend the industry. Budiono, the Vice President of Indonesia from 2009 to 2014, frequently stressed the importance of palm oil in Indonesia’s development (Muhada and Hernanda 2010). According to Bayu Krisnamurthi, who served as Deputy Minister of Agriculture from 2009 to 2011 and Deputy Minister of Trade from 2011 to 2014, producers of rapeseed, sunflower and soybean oils cannot compete with the yield efficiencies of palm oil and thus resort to smear campaigns and protectionism (Lestari 2009). Similar statements are frequently made by high-profile government officials in Indonesia.

The strategic position of palm oil in Indonesia’s economy forces NGOs to brand their actions as being against deforestation rather than in opposition to palm oil. On occasion NGOs even frame their criticisms as an effort to maintain the positive image of Indonesia’s palm oil industry. Bustar Maitar, the then Leader of Greenpeace’s Indonesia Forest Campaign in 2010, acknowledged the importance of this commodity in Indonesia’s economy. He stressed that the Greenpeace campaign is ‘aimed at stopping deforestation and the destruction of peatlands. We demand the government of Indonesia to be firm against major companies that destroy the forests before they further destroy the image of Indonesia’s palm oil’ (Lestari and Sihotang 2010). However, relations between NGOs and the government, especially the Ministry of Agriculture, remain lukewarm. ‘We are open to criticisms, as long as the criticism is valid. It is not valid if one death of an elephant becomes the basis for accusation against all palm oil plantations. NGOs must be objective’, according to Achmad Mangga Barani, the then Director General of Plantations in the Ministry of Agriculture in 2010 (Lestari and Sihotang 2010). The palm oil industry, which is closely aligned with the Ministry of Agriculture, musters everything in its power to strike back against anti-industry campaigns led by NGOs and activists. It appears that one of the reasons for the enactment of Law No. 17/2013 on Societal Organizations was the imposition of stronger restrictions on international NGOs operating in Indonesia, especially those criticizing the palm oil industry such as Greenpeace.

The strategically important palm oil industry tends to unite state actors, though the state does not function in unison. Fragmentation within the state leads to the emergence of different policy networks operating in different parts of the state bureaucracy. In this context, not all government units automatically side with the palm oil industry. The Presidential Unit (UKP4) during President Yudhoyono’s two terms in office (2004 to 2014) was a powerful agency that pushed for reform in the industry and worked closely with domestic and international NGOs. A similar role, but with a different approach, is played by the Presidential Staff Office (KSP) under the current President Joko Widodo. While the Yudhoyono-era UKP4 approached sustainable palm oil production mostly in the context of international norms to protect the environment, and thus engaged in various international schemes such as reducing emissions from deforestation and forest degradation (the REDD project), the Widodo-era KSP focuses more on land conflicts and agrarian reform.

The UKP4 is the agency responsible for the issuance of Presidential Instruction No. 10/2011 on the moratorium on forest conversion, a regulation that was heavily criticized by the palm oil industry. Despite this backlash, the moratorium was extended by President Yudhoyono in 2013 and retained under President Joko Widodo’s administration. UKP4 members intervened in the sector by revising some Ministry of Agriculture regulations, particularly those relating to the issuance of plantation permits (Zuhri 2012). This intervention by government officials antagonized the industry as well as officials from the Ministry of Agriculture, although they never aired their grievances publicly. One interviewee from the palm oil industry stated in 2014 that the UKP4 is an example of how foreign interests can intervene in state affairs through the oversized influence of NGOs. As special agencies under the President, the UKP4 and the KSP are often staffed by practitioners and activists with strong ties to domestic and international NGOs.

Mutually reinforcing factors of state transformation such as fragmentation and the internationalization of the state apparatus were seen in Yudhoyono’s environmental diplomacy. Indonesia’s high-profile environmental diplomacy was on display at the 2015 Paris climate conference when the country became one of the first developing countries to pledge to cut carbon dioxide emissions by 26%. Indonesia’s voluntary national contribution is incongruent with the realities of unsustainable practices on the ground and ever-growing palm oil production targets (Taufik 2016). The contradiction between global pledges and grounded realities illustrates the competition of various social forces to control the domain of national governance. Reformists within the government, many working for the UKP4 (and now the KSP), appear to endorse international norms and commitments.

Through various policy commitments, reformist officials seek to mobilize international resources to support reform initiatives in the forestry sector. Examples include the Australia Forest Carbon Partnership (AU$ 70 million pledged), the German-sponsored emission reduction (REDD) pilot project (EUR 32.4 million), the UN-REDD initiative (US$ 5.6 million), the Korea-sponsored project for the adaptation and mitigation of climate change in forestry (KIPCCF, US$ 5 million) and Australia’s agricultural research fund (ACIAR, US$ 1.4 million) (Muhada and Hernanda 2010). The influence of international funding on the reform of Indonesia’s forestry sector is apparent in the redefinition of the classification of forests following the signing of the Letter of Intent between Indonesia and Norway, in which Norway promised a US$ 1 billion grant for forest conservation (Muhada and Hernanda 2010). National and local resistance is also powerful, however, rendering various policies designed for reform in the forestry sector problematic (Taufik 2016).

Indonesia is the world’s largest producer of palm oil, though the country relies on a relatively inefficient model of expansion rather than land-saving forms of intensification to meet production targets (Varkkey et al. 2018). The pressure for reform from the government, usually from officials with strong international connections, coupled with campaigns by domestic and international NGOs against the palm oil industry, has reduced the opportunity for expansion. The industry resents this development and argues that it decreases the competitiveness of Indonesia’s palm oil production. For instance, Donald Siahaan, a senior researcher in the Palm Oil Research Centre, the industry’s leading commercial research institute, argues that if Indonesia is ‘too focused on responding to environmental issues, other countries will take our market’ (Nurul 2010). The RSPO is perceived negatively in this context because it demands stricter sustainability criteria and less flexible implementation, potentially hindering growth in the palm oil industry. Companies are cautious because withdrawing from the RSPO could damage their business. Consequently, many Indonesian palm oil companies retain their RSPO membership while simultaneously delegitimizing the RSPO. For instance, companies ask the government to protect them against negative campaigns from NGOs that are portrayed as an instrument of the trade war by rival vegetable oil producers from Europe and elsewhere (Choiruzzad 2019).

Conflicting pressures for reform and protection result in the establishment of ambiguous policies such as the ISPO. For reformers, the ISPO provides the opportunity to establish better national governance and management of the palm oil industry. For the industry, the ISPO provides an opportunity to resist the RSPO by delegitimizing it through the presence of a rival scale of governance and certification standard. The ISPO gives industry players more flexibility to expand production. Despite the recent issuance Presidential Regulation No. 44/2020 on Indonesia’s Sustainable Palm Oil Plantation System, there is still no mechanism for independent observers. Furthermore, sensitive issues such as human rights and labour rights remain excluded or only integrated minimally in the ISPO mechanism (Arumingtyas 2020). Having strong networks with national politicians and policymakers, the domestic palm oil industry feels it is advantageous if the governance of the industry remains within the national domain.

The ambiguous nature of the ISPO is illustrated by Suswono, the Minister of Agriculture from 2009 to 2014, when he explained the importance of the ISPO to the palm oil industry. The former minister said that the ISPO ‘is established not to create difficulties for the industry, but to refute environmental-based negative campaigns. The ISPO does not judge one-sidedly. However, all industry players must follow the ISPO’ (Zuhri 2011). While excluding important RSPO concepts such as high conservation value, the ISPO adopts many RSPO principles and criteria, to the extent that there is an ongoing proposal to develop a combined audit mechanism (Suharto et al. 2015). This may simply represent mimicry without any real commitment to implementation, though some reformers within the state see the ISPO as an opportunity to achieve better governance in the sector.

In terms of implementation, the protectionist camp is more prevalent than the reformist camp within Indonesia. It is not surprising that NGOs are sceptical about the ISPO. According to Bustar Maitar, at the time was with Greenpeace Southeast Asia, the intention of the ISPO has always been unclear. He asks whether the ISPO is ‘a serious initiative to develop the palm oil plantation sector without deforestation, or only a gimmick to resist negative campaigns against palm oil’ (Lestari and Hernanda 2010). Companies have their own complaints against the ISPO, arguing that the implementation of the ISPO as a mandatory obligation in 2014 harms the palm oil industry, especially smallholders who account for considerable growth and expansion in the sector. The government lacks the bureaucratic capacity to conduct sustainability assessments, for instance, having limited numbers of certified ISPO auditors. For these reasons, GAPKI demanded that the government postpone the implementation of mandatory ISPO rules in December 2014 (Supriyanto and Hidranto 2014). The government has shown leniency towards the industry. As of 2019, it is estimated that only 4.1 million ha (29.3%) of a nationwide palm oil plantation area measuring 14.03 million ha have been ISPO certified (Anggraini 2019).

Government agencies supported by industry are more aggressive in their use of the ISPO as an instrument to protect the palm oil industry from the perceived trade war with the EU. GAPKI pressures the government to use ISPO standards as a diplomatic instrument to ‘prove’ that Indonesia’s palm oil industry is already sustainable and to refute the accusations and trade barriers against the commodity (GAPKI 2017).^5^ In 2020, this demand was legally accommodated and institutionalized, with the declared aim of ‘increasing the acceptance and competitiveness of Indonesian palm oil products in national and international markets’ included in Presidential Regulation No. 44/2020 on the ISPO system. Despite the efforts of the Indonesian government to promote the ISPO to the international community, it is still not fully accepted by the market. For instance, Vincent Guerend, Ambassador of the European Union to Indonesia and Brunei Darussalam from 2015 to 2019, stated that the ‘ISPO, being implemented only by 15% of Indonesia’s palm oil producers, is not considered as a global standard’ (Murdaningsih 2018). If the ISPO is seen as a watered-down version of the RSPO, then it is unlikely to be taken seriously as a robust standard for certified sustainable palm oil.

Under Joko Widodo’s presidency since 2014, the ambiguity of the ISPO persists. The government is taking measures to strengthen implementation, and yet there are concerns about the commitment and the real motivation of these efforts to strengthen the ISPO. Most recently, in March 2020, the government sought to strengthen ISPO implementation through Presidential Regulation No. 44/2020. Previously, under the Ministry of Agriculture Regulation No. 11/2015, palm oil certification was mandatory for all plantation companies except those producing palm oil for renewable energy and voluntary for plasma or independent smallholders. Under the new regulation in March 2020, all plantation estates and smallholders in the palm oil sector must have ISPO certification. For companies, this mandatory certification requirement took force with immediate effect in March 2020. For smallholders, the government allows up to 5 years for a transition period before ISPO compliance will be enforced. Under the ISPO rules, smallholders are eligible for financial support from national and subnational government to help achieve this transition.

While many NGOs laud this development as a marker of progress in the governance of the palm oil sector, they also demand that the development of new ISPO standards and criteria have more involvement from civil society to ensure that the ISPO is effective in its sustainable business goals. The March 2020 Presidential Regulation includes a specific chapter on how to promote the ISPO to increase its acceptance in international and domestic markets. As part of a complex sustainability agenda, there are signs that the ISPO certification standard is being used to rival the RSPO and to support Indonesian claims of EU discrimination in the vegetable oil trade war (Nathalia 2020; Tyson and Meganingtyas 2020). President Joko Widodo has made palm oil diplomacy a key plank of his foreign policy platform (Choiruzzad 2019), for instance, putting the Ministry of Foreign Affairs in charge of diplomacy, promotion and advocacy of Indonesian palm oil.

The ambiguity surrounding the ISPO is apparent in the broader context of palm oil governance in Indonesia. For instance, Joko Widodo issued Presidential Instruction No. 5/2019 on the Moratorium of Granting New Permits and Improving Governance of Primary Natural Forests and Peatlands. This regulation turned a temporary moratorium of forest conversion subject to extension every 2 years into a permanent rule. In 2020, amidst the COVID-19 pandemic, the government passed a controversial omnibus law on job creation that experts and civil society organization warn will lead to less public scrutiny, undermining sustainability targets (Greenpeace 2020; Mashabi 2020). This situation lends strength to the argument that the ambiguity of the ISPO is a consequence of the meta-phenomenon of Indonesian state transformation in the age of globalization.

## Conclusion

There are persistent technical and political tensions between Southeast Asia’s two major palm oil producers, Indonesia and Malaysia, and the EU directorates and lobbies shaping global environmental and trade standards. This article finds that Indonesia’s fractured responses to transnational private governance in the agricultural sector are connected to continuous processes of state transformation. In the case of palm oil production, the creation of the ISPO certification standard in 2011 is linked to competition among opposing social forces in the context of the internationalization and fragmentation of the Indonesian state. Domestic capital involved in the palm oil industry attempts to counter governance rescaling by transnational actors (mainly companies and NGOs based in developed countries), manifested in the 2004 Roundtable on Sustainable Palm Oil (RSPO), in order to sustain their perceived privilege in the national scale of governance. Indonesian trade associations and industry players exploit their connections with relevant government agencies such as the Ministry of Agriculture and the Ministry of Trade. At the same time, reformists within the state bureaucracy internalize global norms of sustainability and attempt to push reforms in the governance of the palm oil industry. This jockeying for power and influence resulted in the establishment the ISPO, an ambiguous policy that internalizes RSPO standards and mimics global and EU norms while simultaneously creating a rival form of governance to protect the palm oil industry from external pressures.

The inherent ambiguity of the ISPO causes practical dilemmas on two fronts. The ISPO is generally ineffective in its implementation, to the disappointment of reformists within the Indonesian state, and at the same time is unable to serve as a legitimate alternative to the RSPO in the global market, to the disappointment of protectionists. The low level of acceptance of the ISPO by international markets explains why Indonesian palm oil companies tend to remain in the RSPO despite their criticisms of membership, representation and the interventionist nature of transnational private governance.

Active campaigning by industry players with close political connections has elevated palm oil to the status of a strategic priority in Indonesian foreign policy. In October 2019, for instance, during her first speech as Indonesia’s Foreign Minister, Retno Marsudi specifically mentioned palm oil as a diplomatic priority. The foreign minister contends that palm oil is a strategic national commodity and the growth of the sector ‘is a fundamental matter because it is related to the livelihoods of more than 16 million people, especially small-scale farmers and their families’ (CNN Indonesia 2019). The authors anticipate that the jostling between transnational and national domains of governance will continue to intensify, and the evolving power dynamic will be shaped by simultaneous and overlapping competition between social forces that are advocating palm oil policies at different scales of governance.

## Data Availability

Not applicable.

## Footnotes

1 There are now RSPO-RED voluntary requirements to improve compliance with the EU Renewable Energy Directive. See https://rspo.org/certification/rspo-red.

2 The ISPO was established through Ministry of Agriculture Regulation No. 19/Permentan/OT.140/3/2011.

3 The film *Asimetris* is available for public view at https://www.youtube.com/watch?v=2OhaxAalJdk.

4 As of December 2020, the top ten RSPO countries by membership are the USA, Germany, the UK, Italy, the Netherlands, Japan, France, China, Belgium and Spain.

5 Similar developments can be observed under the Malaysian Sustainable Palm Oil (MSPO) scheme. The MSPO was launched as a voluntary government-regulated scheme in 2014 and became mandatory in 2017. With strong industry backing, Malaysian government agencies have pushed for, and received, recognition of the MSPO in other major export markets, for instance, from the Tokyo 2020 Olympics and Paralympics Games Sustainable Sourcing Code Committee, China’s Green Food Development Centre, and the Solvent Extractors’ Association of India, as a form of pushback against developments in the EU.
